# Supplementary material for: Genome-wide association analysis and pathway enrichment provide insights into the genetic basis of photosynthetic responses to drought stress in Persian walnut
Source: Hortic Res. 2022 Jun 7;9:uhac124. doi: 10.1093/hr/uhac124 (PMC9343916; doi:10.1093/hr/uhac124)
Supplement: Web_Material_uhac124 [file web_material_uhac124.zip › Supplementary Material_Revise.docx]

**Genome-wide association analysis and pathway enrichment provide insights into the genetic basis of photosynthetic responses to drought stress in Persian walnut**

 Mohammad M. Arab^1^, Patrick J. Brown^2^, [Rostam Abdollahi-Arpanahi](http://scholar.google.com/citations?user=OmDA_YQAAAAJ&hl=en)^3^, Seyed Sajad Sohrabi^4^, Hossein Askari^5^, Sasan Aliniaeifard^6^, Ali Mokhtassi-Bidgoli^7^, Mohsen B. Mesgaran^2^, Charles A. Leslie^2^, Annarita Marrano^2^, David B. Neale^2^ and [Kourosh Vahdati](http://scholar.google.com/citations?user=ZPuaA8kAAAAJ&hl=en)^1^*

^1^Department of Horticulture, College of Aburaihan, University of Tehran, Tehran, Iran

^2^Department of Plant Sciences, University of California, Davis, CA 95616

^3^Department of Animal and Dairy Science, University of Georgia, Athens, GA, USA

^4^ Department of Plant Production and Genetic Engineering, Faculty of Agriculture, Lorestan University, Khorramabad, Iran

^5^ Department of Plant Sciences and Biotechnology, Faculty of Life Sciences and Biotechnology, Shahid Beheshti University, Tehran, Iran

^6^Photosynthesis laboratory, Department of Horticulture, College of Aburaihan, University of Tehran, Tehran, Iran

^7^Department of Agronomy, Faculty of Agriculture, Tarbiat Modares University, Tehran, Iran

^*^Corresponding Authors: Kourosh Vahdati (email: kvahdati@ut.ac.ir)

**Author mails respectively:**

[mm.arab@ut.ac.ir](mailto:mm.arab@ut.ac.ir), [pjbrown@ucdavis.edu](mailto:pjbrown@ucdavis.edu), [rostam.abdollahi@uga.edu](mailto:rostam.abdollahi@uga.edu), [sohrabi.sa@fa.lu.ac.ir](mailto:sohrabi.sa@fa.lu.ac.ir), askarihossein@yahoo.com, [aliniaeifard@ut.ac.ir](mailto:aliniaeifard@ut.ac.ir), [mokhtassi@modares.ac.ir](mailto:mokhtassi@modares.ac.ir), [mbmesgaran@ucdavis.edu](mailto:mbmesgaran@ucdavis.edu), caleslie@ucdavis.edu, [amarrano@ucdavis.edu](mailto:amarrano@ucdavis.edu), [dbneale@ucdavis.edu](mailto:dbneale@ucdavis.edu), [kvahdati@ut.ac.ir](mailto:kvahdati@ut.ac.ir)

***Corresponding author:** [kvahdati@ut.ac.ir](mailto:kvahdati@ut.ac.ir)

Kourosh Vahdati

Professor, Department of Horticulture, College of Aburaihan,

University of Tehran (UT), Tehran, Iran

Tel: +9821 36041089

Fax: +9821 36041089

**This PDF file includes:
Supplementary Figure S1-S14
Supplementary Tables S1-S8
(Supplementary Datasets S1-S7 are provided in separate excel files)**

**Material and Methods:**

**Seed germination and seedling production**

Open-pollinated seeds were collected from 150 mother trees (seed family) locally-adapted across Iran in 2015. Before sowing, walnut seeds were exposed to running water for 10 days, treated with a fungicide (Captan 5%), and subsequently stratified in a refrigerator at 4°C for 4–6 weeks to break dormancy^1^. Twenty seeds per mother tree (family) were then sown in 7 l polyethylene pots containing a soil, sand, and leaf manure mixture and care was taken to avoid over compaction of the soil. Each pot had three holes at the bottom for imposing controlled stress. Seedlings were grown in the greenhouse at 25/20 °C (day/night), a 16 h photoperiod, and 50 (±10)% relative humidity under normal day light, while continuously irrigated. From these 150 half-sib families, a total of 1500 plants were produced (at least 10 plants per family), of which 6–8 uniform plants from each family were selected for the drought stress experiment in the first year. After the first year, prior to starting the second year of experiment, plants were transferred to 15 litre pots containing a mixture of soil, sand, and manure (2:1:1, v/v/v). Pots were then arranged in a greenhouse as described by Arab et al. (2020)^2^. For each half-sib family, at least two or three plants per treatment (normal and drought) were phenotyped for photosynthesis-related traits during the two years drought stress experiments.

**Drought score index and** **relative water content (RWC)**

Score index was visually graded on a range of 1 to 9 according to the appearance characteristics of the plant. Plants rated 1 were absolutely healthy plants with dark green leaves, while a rate of 9 indicates dried plants with necrotic leaves. Leaf discs were collected from fully expanded leaves of each plant. Ten uniform leaf discs were weighed immediately to compute the fresh weight (FW), and submerged in 25-ml closed tubes filled with distilled water for 24 h at 4^◦^C in darkness and again weighed to obtain turgor weight (TW). The leaf discs were then dried in an oven at 70^◦^C for 24 h, and the dry weight (DW) was recorded. Finally, the RWC or relative turgidity of each sample was calculated as RWC = [(FW–DW)/(TW–DW)] × 100. These traits were measured 5 times during the two years of drought stress experiments.

**Chlorophyll fluorescence measurements (OJIP-test)**

Using the OJIP-test approach, several phenomenological and biophysical parameters quantifying the PSII and PSI behaviours have been calculated. Briefly, the OJIP steps measured indicate fluorescence intensities at 50 μs referred to as F_0_ (minimum fluorescence intensity) and occurs at the O step when all PSII reaction centres are open. The leaflet samples were immediately exposed to a saturating light of ~3000 μmol m^−2^ s^−1^ , and fluorescence intensity at 2 ms (J-step, F_J_), the intensity at 30 ms (I-step, F_I_), and the maximum fluorescence intensity (approximately 200 ms, F_M_) when all PSII RCs are closed (P step) were collected to be used for further calculations. In addition, other OJIP parameters were calculated according to the methods given by Strasser et al., (2004)^3^ to detect the injury site on the electron acceptor site of photosystem II (PSII). The other parameters were calculated as follows: the performance index (PI_ABS_), PSII maximum photochemical efficiency (F_V_/F_M_), maximum quantum yield of energy trapping by PSII (φ_Po_), quantum yield of intersystem electron transport (φ_Eo_), PSII antenna size (ABS/RC), trapped energy flux (TR_0_/RC), electron transport flux per reaction center (ET_0_/RC), reduction of PSI end electron acceptors per reaction center (RE_0_/RC), and dissipated energy flux (DI_0_/RC) were recorded to provide more structural information on the photosynthetic apparatus (Strasser et al. 2000, 2004).

### **Genotyping with the Axiom *J. regia* 700K SNPs array, and quality control**

The 95 mother trees were genotyped at 609,658 SNPs evenly distributed throughout the walnut genome using the high-density walnut array from Affymetrix^4^ as described by Arab et al (2019)^5^. Briefly, approximately 50 µL (15 ng µL^–1^) of high-quality walnut genomic DNA per each sample from each individual was sent to Affymetrix (now part of Thermo Fisher Scientific, Santa Clara, CA, USA; [www.affymetrix.com](http://www.affymetrix.com)) for genotyping. SNP calling was performed by the Bioinformatics Core of Affymetrix using the Axiom Best Practices Genotyping Workflow with default walnut SNP call rate cut-off = 97%, and DQC cut-off = 0.82. After genotype calling, quality control was applied using PLINK v1.9 software^6^, and a total of 299,536 high-quality SNPs of Poly High Resolution (PHR) class with minor allele frequency (MAF) above 5% and missing rate below 20% remained for the following genome-wide analysis.

### **Population structure analysis**

Three complementary approaches including PCA, fastStructure, and Neighbor-Joining (NJ) were used to assess population structure. The results of genetic structure analysis of 95 mother trees genotyped through the new Axiom™ J. regia 700K SNP array have already been described by Arab et al (2019)^5^. To study the genetic structure of mother trees and their offspring that were genotyped through GBS, we first run a PCA analysis using the R package GAPIT v3.0^7^. In addition, to determine the number of genetic clusters, a Bayesian clustering approach using fastStructure^8^ was applied. To confirm further the number of clusters inferred by the population structure analysis, Neighbor-Joining (NJ) phylogenetic tree was also constructed using GAPIT.

**Results:**

**Candidate gene identification for significant SNPs**

The annotation results revealed that significant SNPs located within the genes were related to [phosphoenolpyruvate carboxykinase (ATP) activity](https://www.ebi.ac.uk/QuickGO/term/GO:0004612), abscisic acid (ABA) signaling pathways, water channels (Aquaporins), light control of development, cutin synthesis, cell expansion and the suggestive SNPs fell in genes that are involved in light receptor (light-harvesting complex), [sugar mediated signaling pathway](https://www.ebi.ac.uk/QuickGO/term/GO:0010182), biosynthesis of membrane lipids, and antioxidant defense mechanism. Our results also demonstrated that identified genes around the significant SNPs (20 K) were related to the chloroplast development, trans-membrane electron transporter, electron flow toward the plastoglobule plastoquinone pool, abscisic acid (ABA) signaling pathway, Phytochrome A1, and the identified genes around the suggestive SNPs are involved in the exchange of ADP and ATP across the mitochondrial inner membrane, signal transduction pathways, [proton transmembrane transport](https://www.ebi.ac.uk/QuickGO/term/GO:1902600), the flow of protons through the CF_0_ complex, plastoquinol—plastocyanin reductase ([cytochrome b6f complex](https://en.wikipedia.org/wiki/Cytochrome_b6f_complex)). The remaining genes were involved in the regulation of photosynthesis and drought tolerance.

**References:**

1. Vahdati, K. *et al.* Screening for drought-tolerant genotypes of Persian walnuts (*Juglans regia* L.) during seed germination. **44**, 1815-1819 (2009).

2. Arab, M.M. *et al.* Combining phenotype, genotype, and environment to uncover genetic components underlying water use efficiency in Persian walnut. **71**, 1107-1127 (2020).

3. Strasser, R.J., Tsimilli-Michael, M. & Srivastava, A. Analysis of the chlorophyll a fluorescence transient in Chlorophyll a fluorescence 321-362 (Springer, 2004).

4. Marrano, A. *et al.* A new genomic tool for walnut (*Juglans regia* L.): development and validation of the high‐density Axiom™ *J. regia* 700K SNP genotyping array. **17**, 1027-1036 (2019).

5. Arab, M.M. *et al.* Genome-wide patterns of population structure and association mapping of nut-related traits in Persian walnut populations from Iran using the Axiom *J. regia* 700K SNP array. **9**, 1-14 (2019).

6. Purcell, S. *et al.* PLINK: a tool set for whole-genome association and population-based linkage analyses. **81**, 559-575 (2007).

7. Lipka, A.E. *et al.* GAPIT: genome association and prediction integrated tool. **28**, 2397-2399 (2012).

8. Raj, A., Stephens, M. & Pritchard, J.K. fastSTRUCTURE: variational inference of population

structure in large SNP data sets. Genetics 197, 573-589 (2014).

| **First Experiment (September 2016)** | | | |
| --- | --- | --- | --- |
| **Year 1** | **Plant Martials** | 150 families (1500 six-month-old seedling planted on 7L pot) | |
|  | **Time 1 (11)** | **Moderate water-stress**  **Three weeks after the start of the experiment** | |
|  |  | **Well-watered group**  **WW11**  **(SMC above 75% FC)** | **Water-stressed group**  **WS11**  **(SMC** ∼**40-50% FC)** |
|  |  |  | |
|  | **Time 2 (12)** | **Severe water-stress**  **Five weeks after the start of the experiment** | |
|  |  | **Well-watered group**  **WW12**  **(SMC above 75% FC)** | **Water-stressed group**  **WS12**  **(SMC** ∼**25-35% FC)** |
| **Second Experiment (May 2017)** | | | |
| **Year 2** | **Plant Martials** | 150 families (1200 Fifteen-month-old seedling planted on 15L pot) | |
|  | **Time 1**  **(21)** | **Severe water-stress**  **24 days after the start of the experiment** | |
|  |  | **Well-watered group**  **WW21**  **(SMC above 75% FC)** | **Water-stressed group**  **WS21**  **(SMC** ∼**25-35% FC)** |
|  |  |  | |
|  | **Time 2**  **(22)** | **Recovery**  **Two weeks after re-watering** | |
|  |  | **Well-watered group**  **WW22**  **(SMC above 75% FC)** | **Re-watering group**  **WR22**  **(SMC above 75% FC)** |

**Supplementary Figure S1.** A schematic diagram of applying water stress on Persian walnut populations. SMC: soil moisture content, FC: field capacity of soil.


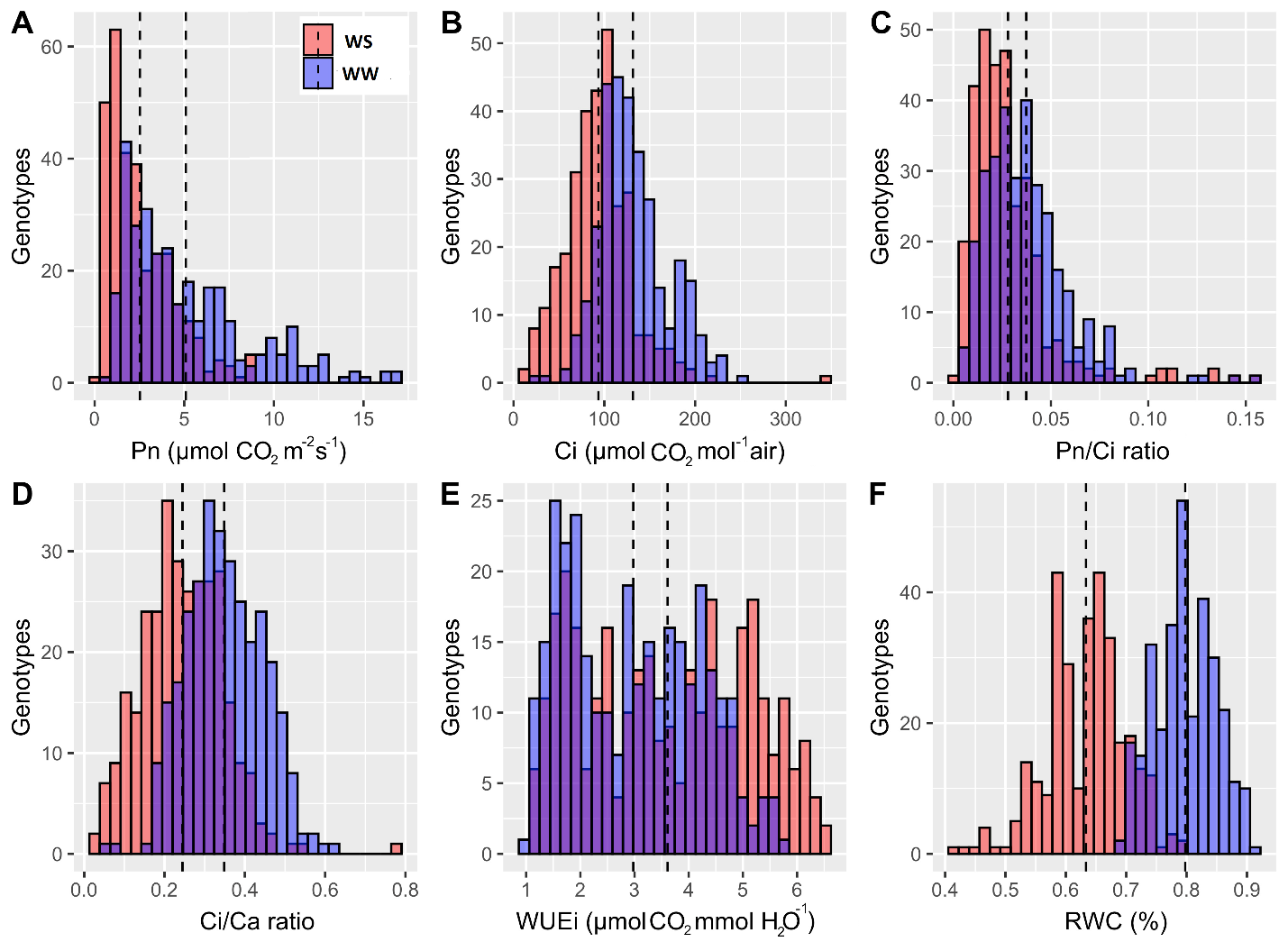


**Supplementary Figure S2.** Distribution of the photosynthetic related traits of fifteen-month-old walnut plants under well-watered (blue), water-stress (red) conditions and overlap between them (purple) in the second year of experiment. (a) P_n_, (b) C_i_, (c) P_n_/C_i_, (d) C_i_/C_a_, (e) WUE_i_, and (f) RWC in the 140 walnut families. Traits are indicated on the x‐axis and number of lines on the y‐axis. Dashed vertical lines indicate the mean of each distribution.


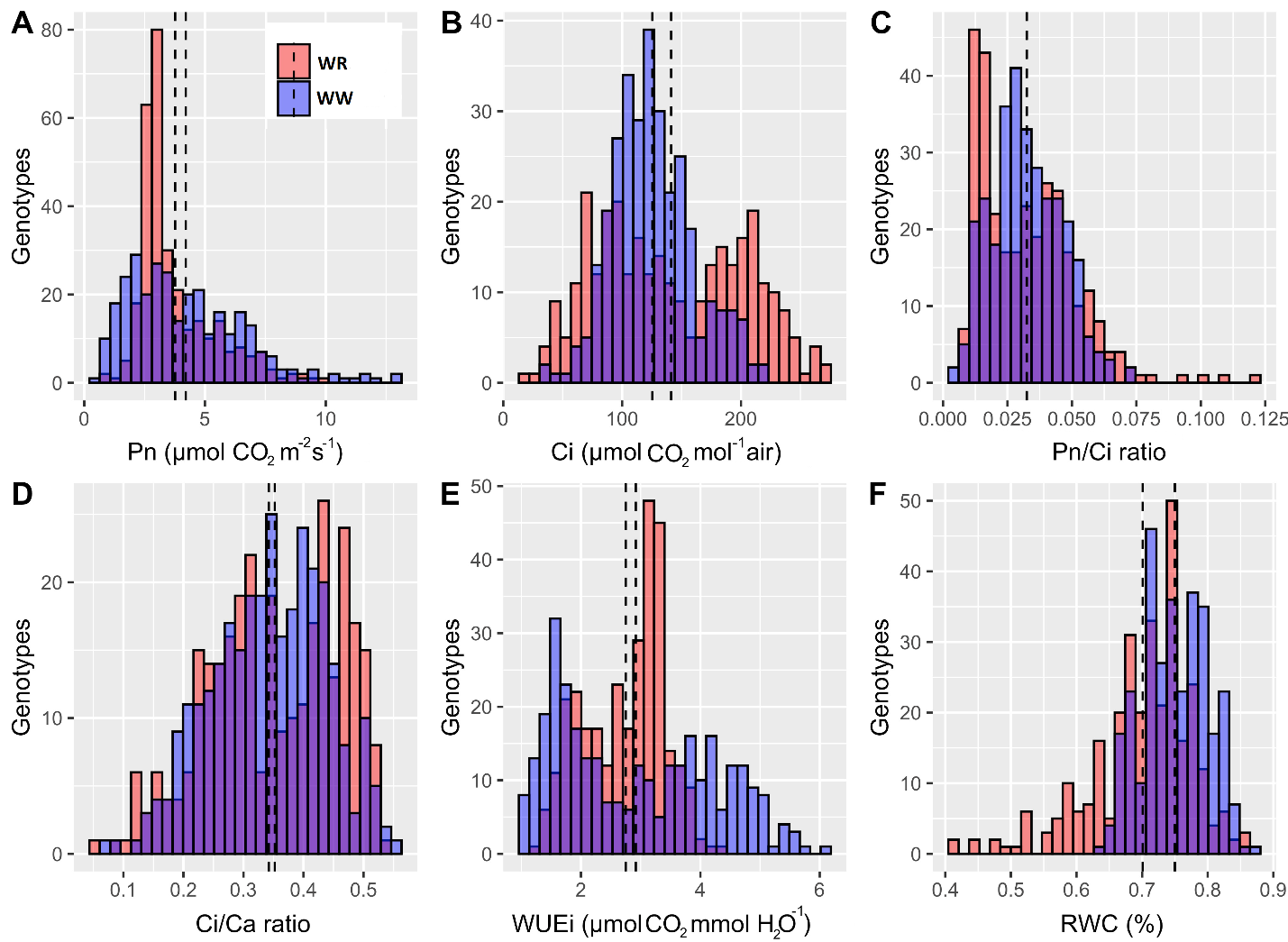


**Supplementary Figure S3.** Distribution of the photosynthetic related traits of fifteen-month-old walnut plants under well-watered (blue), water-recovery (red) conditions and overlap between them (purple) in the second year of experiment. (a) P_n_, (b) C_i_, (c) P_n_/C_i_, (d) C_i_/C_a_, (e) WUE_i_, and (f) RWC in the 140 walnut families. Traits are indicated on the x‐axis and number of lines on the y‐axis. Dashed vertical lines indicate the mean of each distribution.


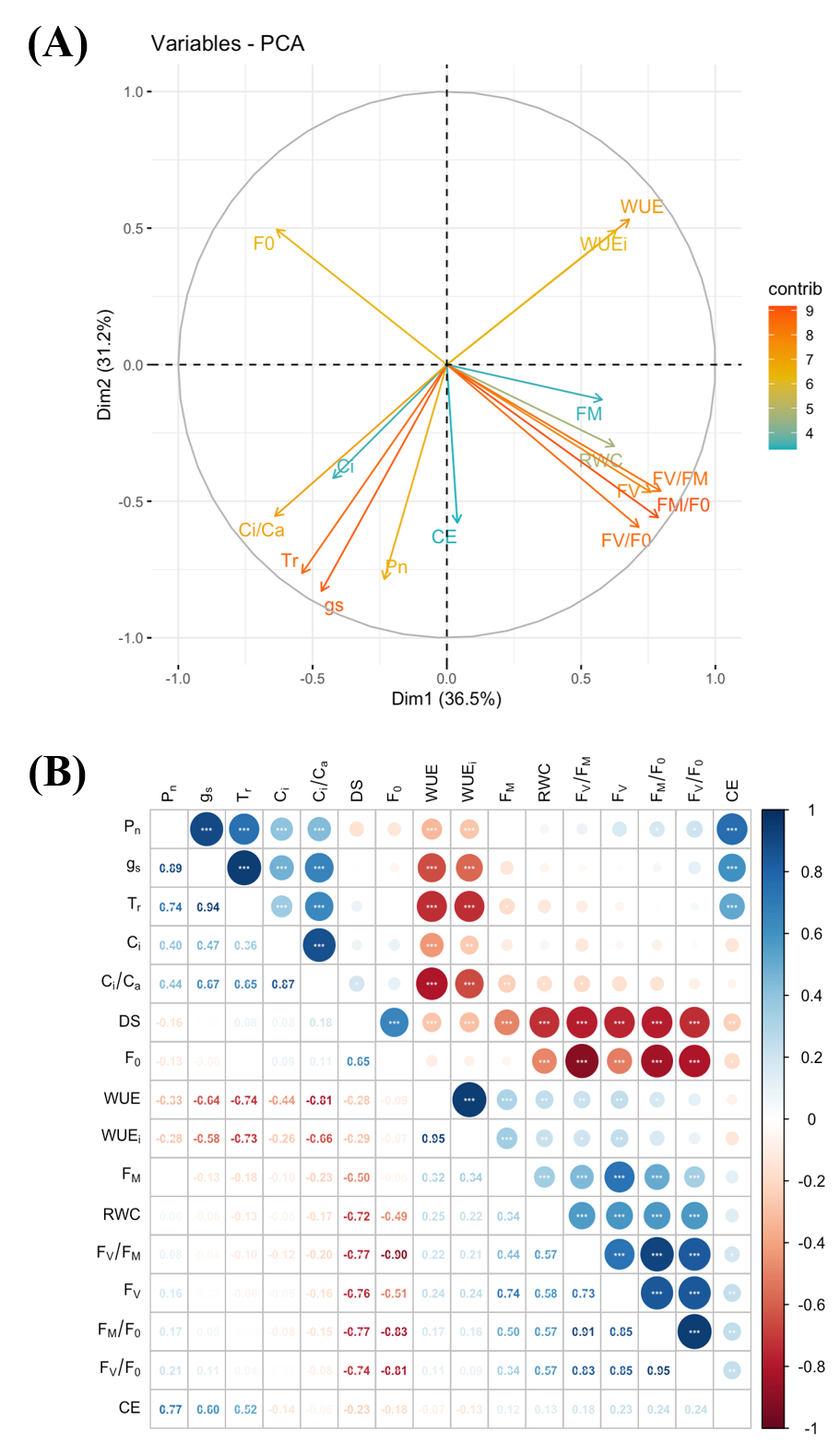


**Supplementary Figure S4.** Principal component analysis (PCA) and correlation coefficient plots of photosynthetic traits in the 140 walnut families grown in a common garden under severe water stress in the first year of experiment. The size of the circle is proportional to the strength of the correlation coefficients. The colour spectrum, bright blue to bright red represents highly positive to highly negative correlations. Stars in circle indicate the significance of correlations (*P ≤ 0.05, **P ≤ 0.01, and ***P ≤ 0.001). See Table 1 for the definition of measured traits.


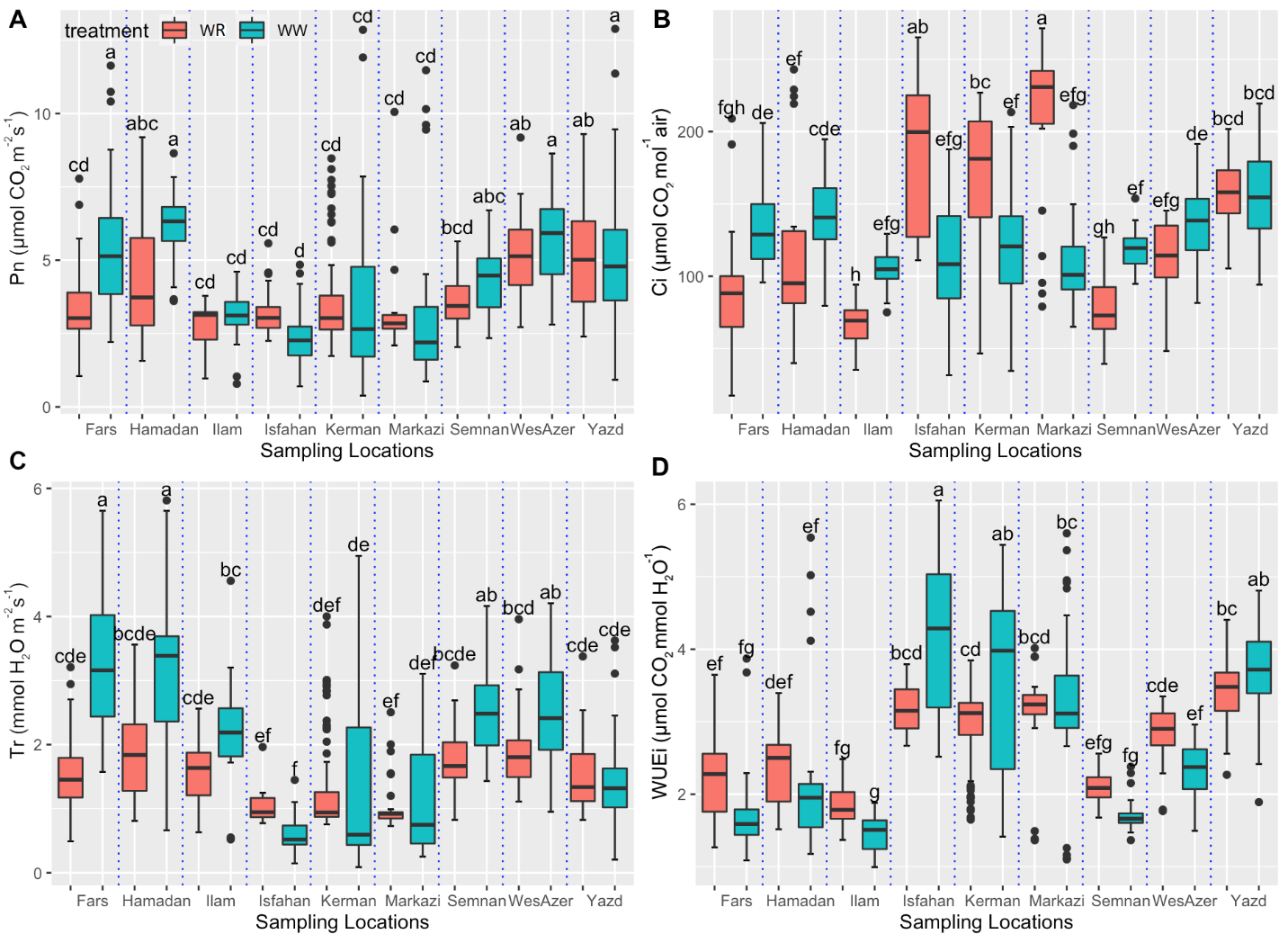


**Supplementary Figure S5.** Average performance of the photosynthetic related traits in the 140 walnut families grown in a common garden under well-water and water recovery conditions in the first-year experiment. WW: well-watered; WR: water recovery. Different letters indicate statistically significant differences at the level of p < 0.01 (Tukey's test).


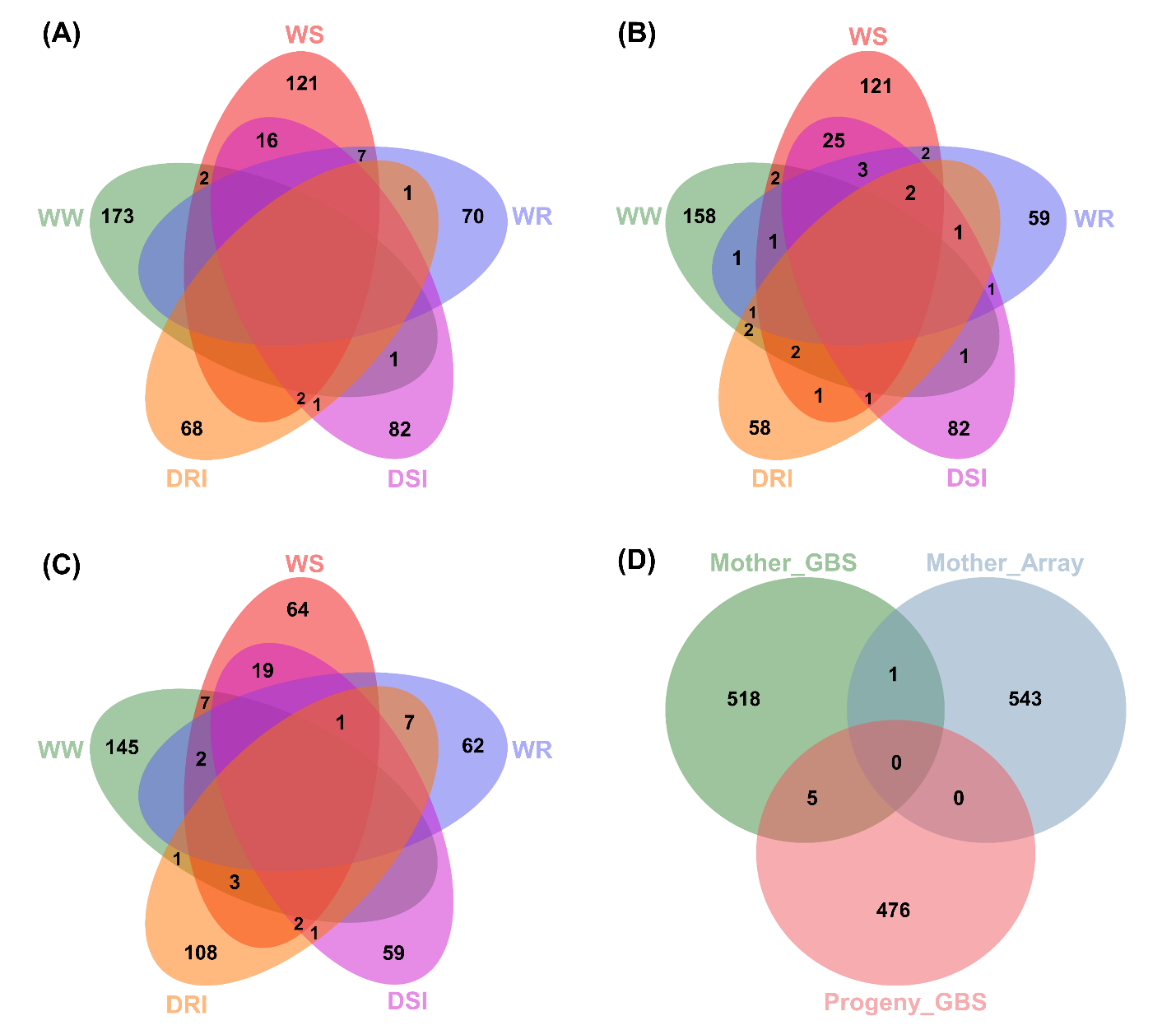


**Supplementary Figure S6.** Venn diagrams depicting the suggestive SNPs identified for all the photosynthetic traits across experiments (First and second years) and different conditions through GWAS using different datasets MArray (A), MGBS (B) and PGBS (C) and highlight the concordance between different datasets (D). Numbers of associations are arranged in circles across different conditions (A, B, and C), and circle overlaps representing numbers of SNPs associated with more than one genotyping method category (D).


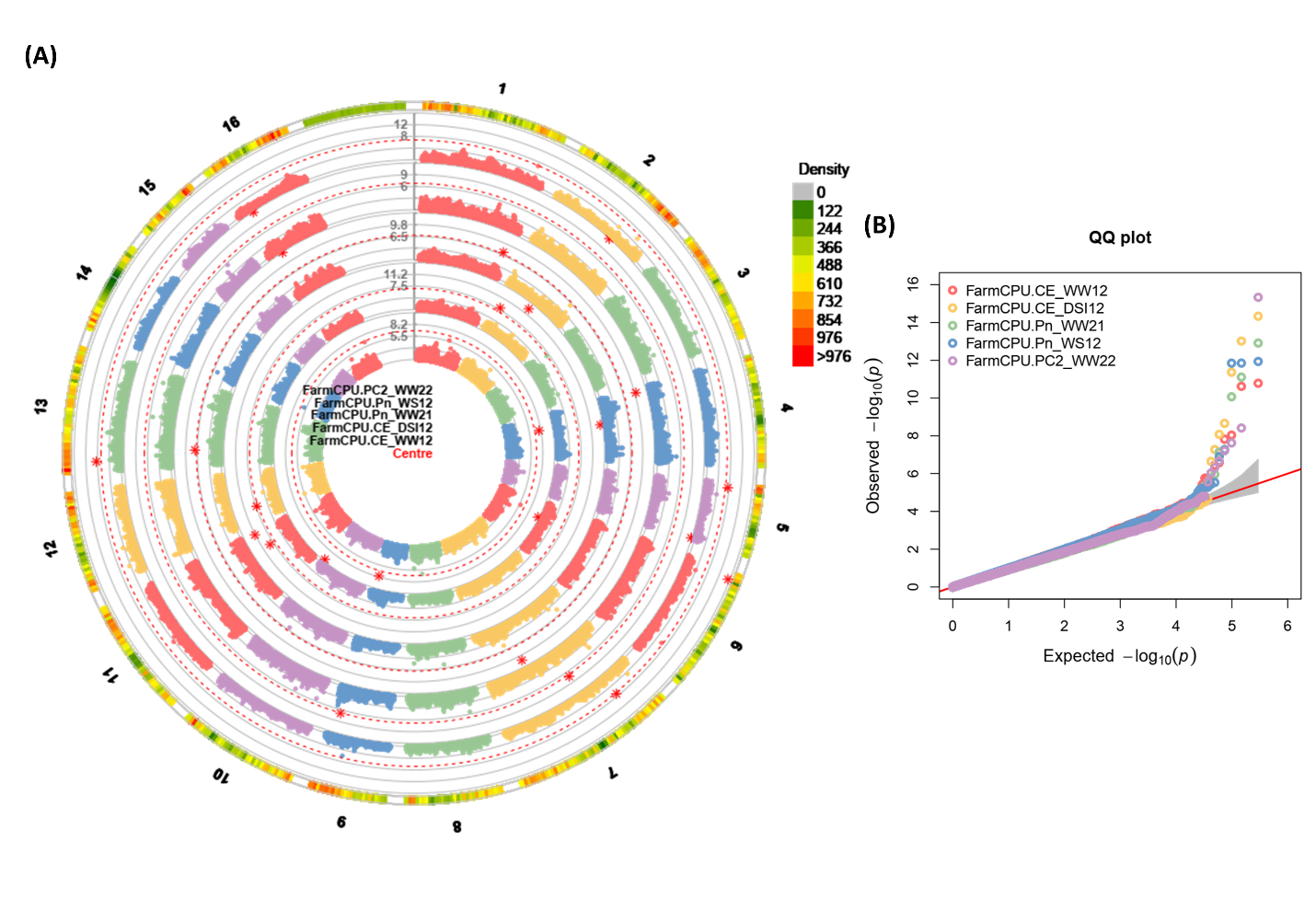


**Supplementary Figure S7**. (A) and (B) Circular Manhattan plots (left), and quantile-quantile plots (right) of association analysis using the MArray dataset and FarmCPU (Q *+* K) model for gas-exchange related traits from centre to the outside of plot including; (a) CE_WW12; (b) CE_DSI12, (c) Pn_WW21, (d) Pn_WS12, and (e) PC2_WW22. The circles of red dashed lines represent the Bonferroni-corrected significance threshold. Red stars indicate genome-wide significant findings. The numbers and colored boxes around the outermost circle indicate the chromosome and the density of single-nucleotide polymorphisms (SNPs) where green to red represent lower to higher density. For QQ plots, X-axis represents expected − log_10_ (*p-value*) and Y-axis is observed − log_10_ (*p-value*) of each SNPs. Well-water: WW, water-stress: WS, drought stress index: DSI. 12: severe drought stress in the first year, 21: severe drought stress in the second year, and 22: recovery condition in the second year.


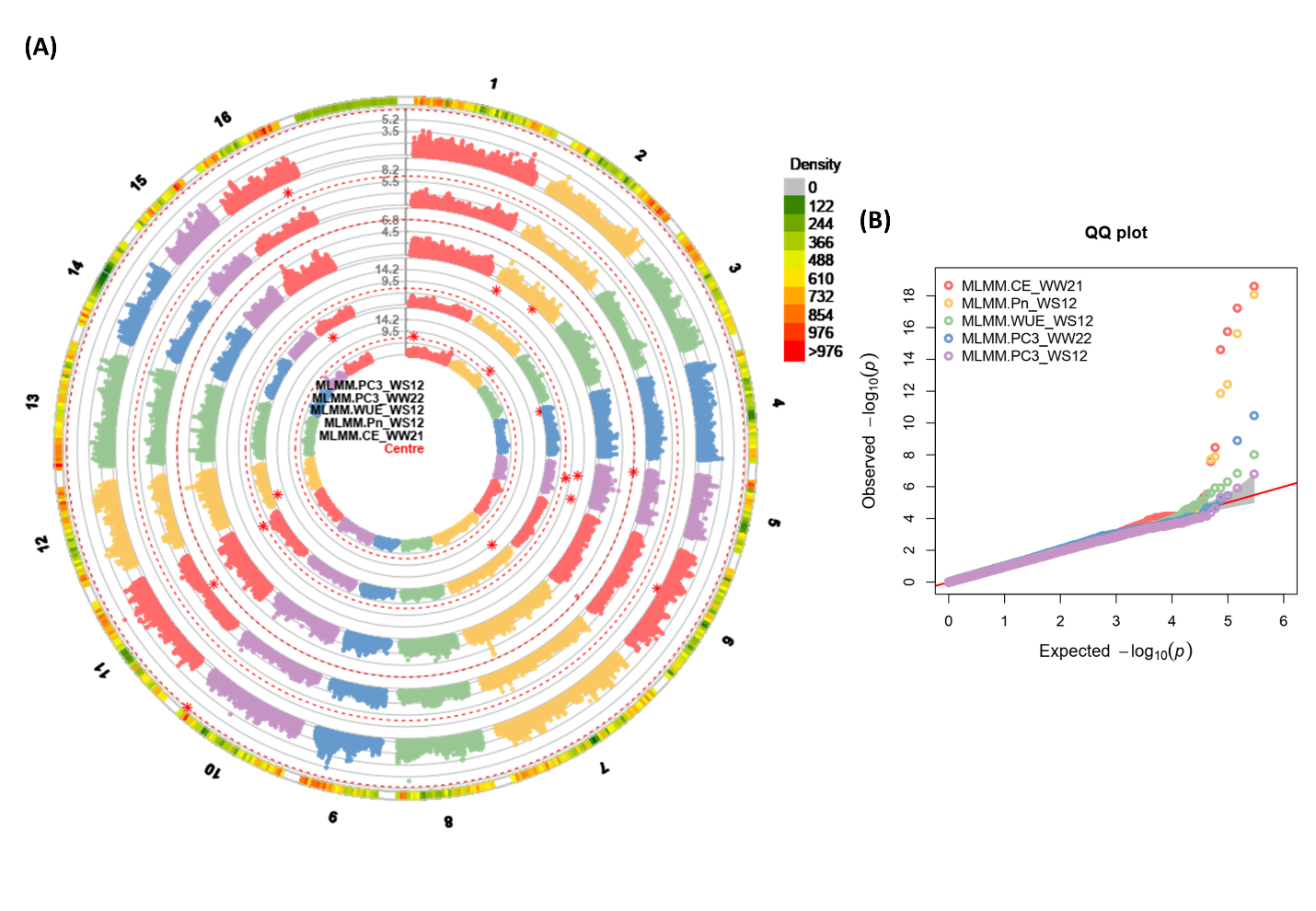


**Supplementary Figure S8**. (A) and (B) Circular Manhattan plots (left), and quantile-quantile plots (right) of association analysis using the the MArray dataset and MLMM (Q *+* K) model for gas-exchange related traits from centre to the outside of plot including; (a) CE_WW21, (b) Pn_WS12, (c) WUE _WS12, (d) PC3_WW22 and (e) PC3_WS12. The circles of red dashed lines represent the Bonferroni-corrected significance threshold. Red stars indicate genome-wide significant findings. The numbers and colored boxes around the outermost circle indicate the chromosome and the density of single-nucleotide polymorphisms (SNPs) where green to red represent lower to higher density. For QQ plots, X-axis represents expected − log_10_ (*p-value*) and Y-axis is observed − log_10_ (*p-value*) of each SNPs. Well-water: WW, water-stress: WS. 12: severe drought stress in the first year, 21: severe drought stress in the second year, and 22: recovery condition in the second year


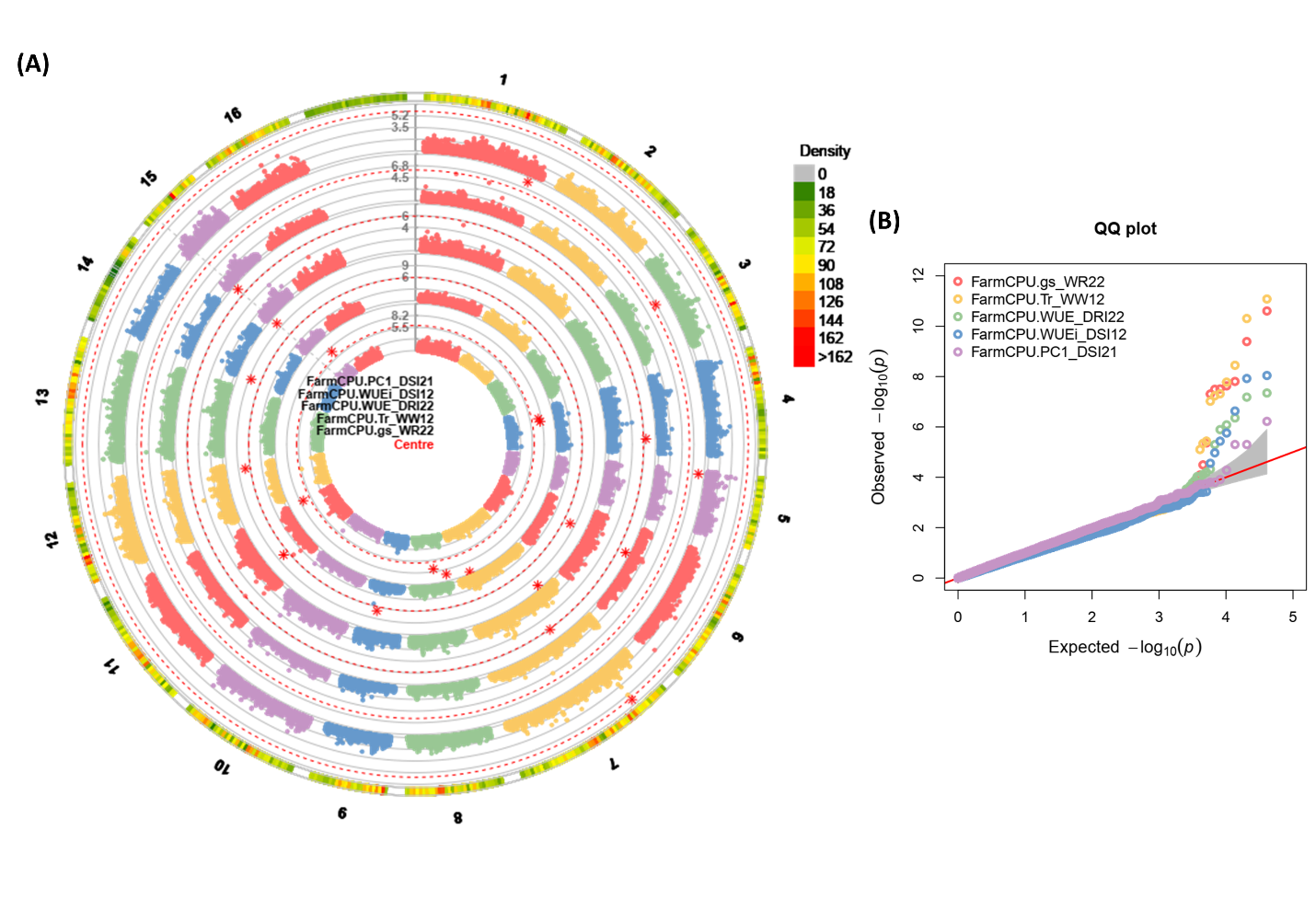


**Supplementary Figure S9**. (A) and (B) Circular Manhattan plots (left), and quantile-quantile plots (right) of association analysis using the MGBS dataset and FarmCPU (Q *+* K) model for gas-exchange related traits from centre to the outside of plot including; (a) gs_WR22, (b) T_r__WW12, (c), WUE_DRI22,(d), WUE_i__DSI12, and (e) PC1 _DSI21. The circles of red dashed lines represent the Bonferroni-corrected significance threshold. Red stars indicate genome-wide significant findings. The numbers and colored boxes around the outermost circle indicate the chromosome and the density of single-nucleotide polymorphisms (SNPs) where green to red represent lower to higher density. Vertical grey dashed lines are drawn through GWAS findings to indicate multi-trait associations. For QQ plots, X-axis represents expected − log_10_ (*p-value*) and Y-axis is observed − log_10_ (*p-value*) of each SNPs. Well-water: WW, water-recovery: WR, drought stress index: DSI and drought recovery index: DRI. 12: severe drought stress in the first year, 21: severe drought stress in the second year, and 22: recovery condition in the second year


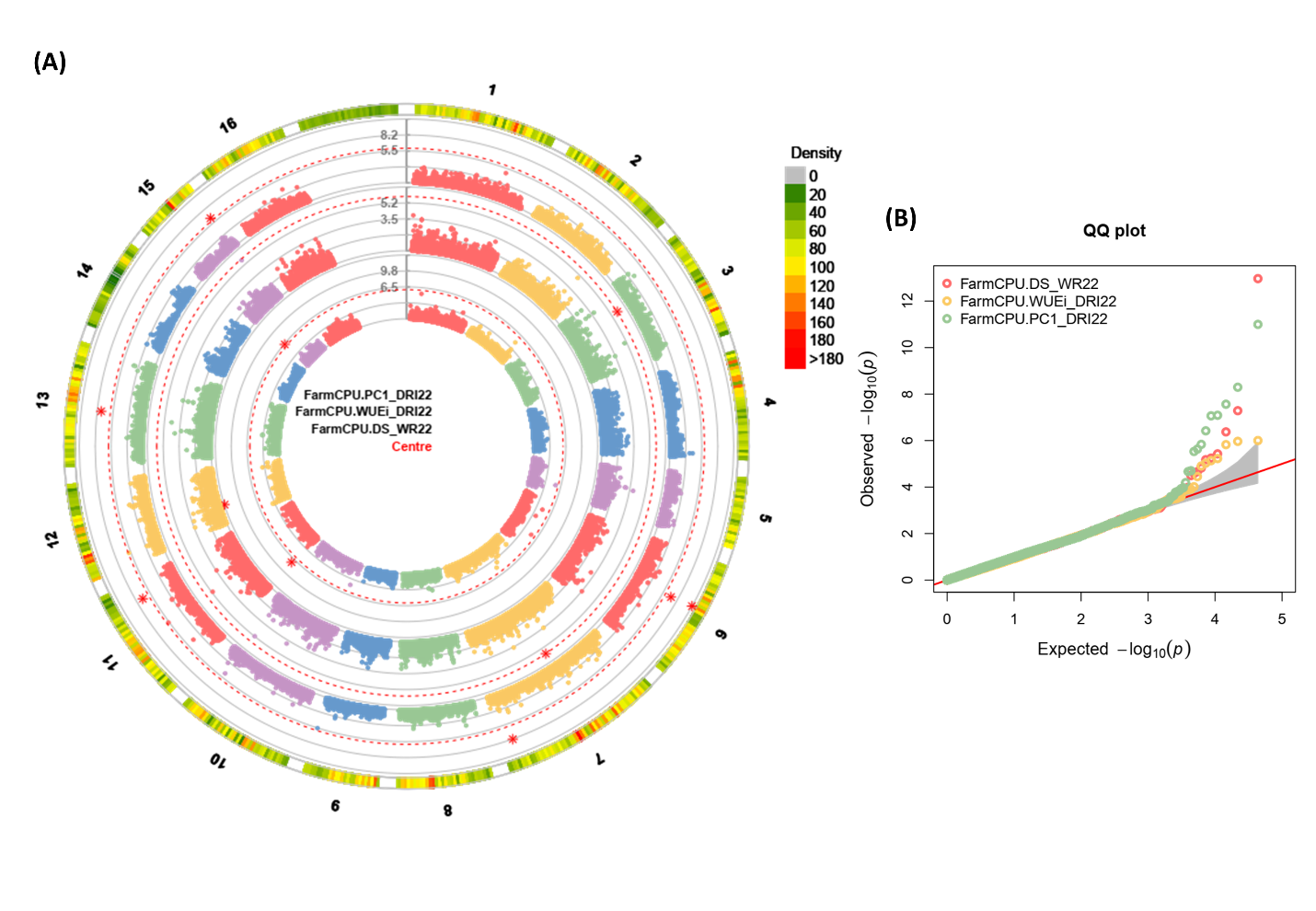


**Supplementary Figure S10**. (A) and (B) Circular Manhattan plots (left), and quantile-quantile plots (right) of association analysis using the PGBS dataset and FarmCPU (Q *+* K) model for gas-exchange related traits from centre to the outside of plot including; (a) DS_WR22, (b) WUEi_DRI22, and (c) PC1 _DRI22. The circles of red dashed lines represent the Bonferroni-corrected significance threshold. Red stars indicate genome-wide significant findings. The numbers and colored boxes around the outermost circle indicate the chromosome and the density of single-nucleotide polymorphisms (SNPs) where green to red represent lower to higher density. For QQ plots, X-axis represents expected − log_10_ (*p-value*) and Y-axis is observed − log_10_ (*p-value*) of each SNPs. Water-recovery: WR, and drought recovery index: DRI. 22: recovery condition in the second year.

**
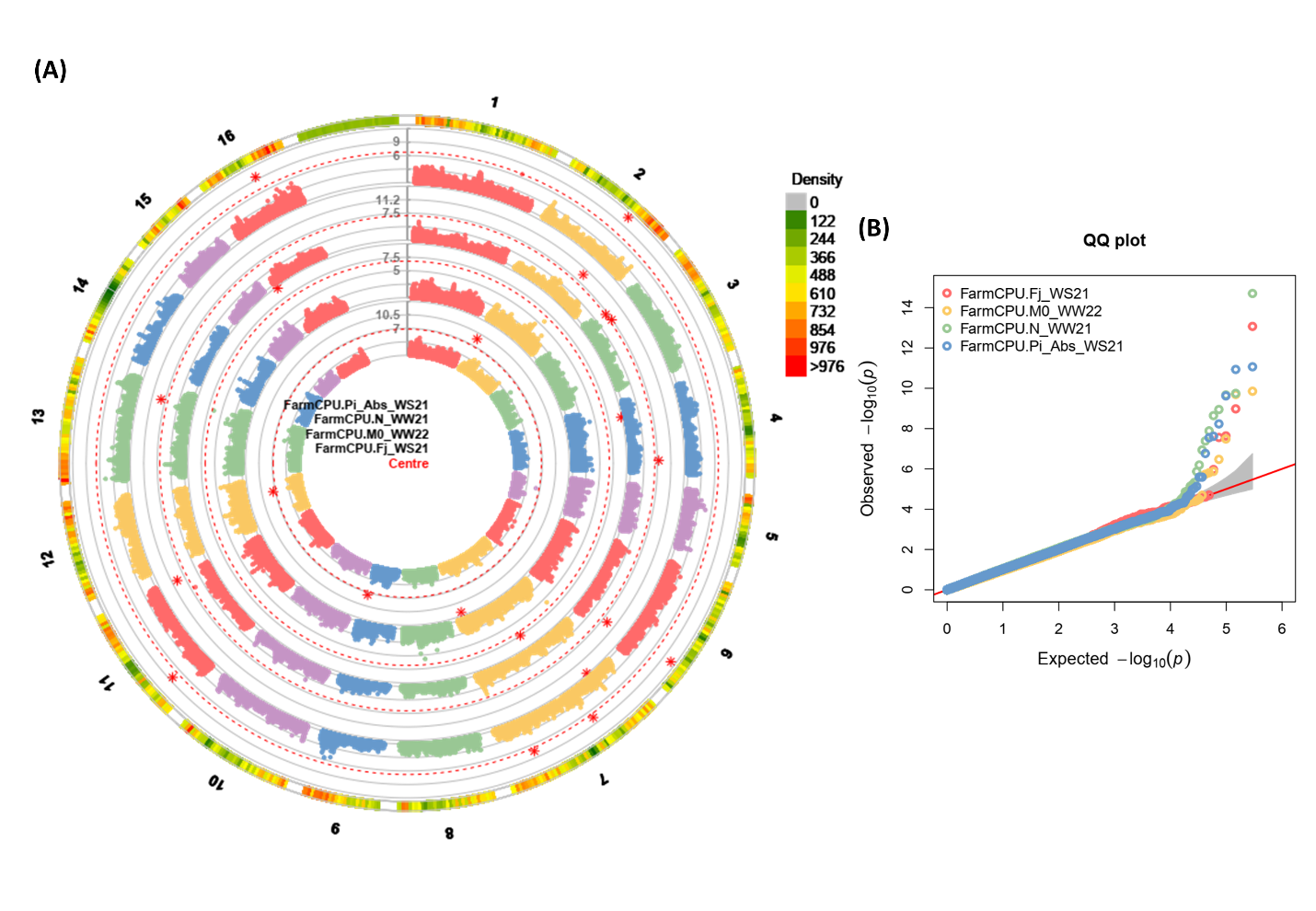
**

**Supplementary Figure S11.** (A) and (B) Circular Manhattan plots (left), and quantile-quantile plots (right) of association analysis using the MArray dataset and FarmCPU (Q *+* K) model for chlorophyll fluorescence related traits from centre to the outside of plot including; (a) F_J__WS21, (b) M_0__WW22, (c) N_WW21, and (d) PI_ABS__WS21. The circles of red dashed lines represent the Bonferroni-corrected significance threshold. Red stars indicate genome-wide significant findings. The numbers and colored boxes around the outermost circle indicate the chromosome and the density of single-nucleotide polymorphisms (SNPs) where green to red represent lower to higher density. For QQ plots, X-axis represents expected − log_10_ (*p-value*) and Y-axis is observed − log_10_ (*p-value*) of each SNPs. Well-water: WW, water-stress: WS. 21: severe drought stress in the second year, and 22: recovery condition in the second year.


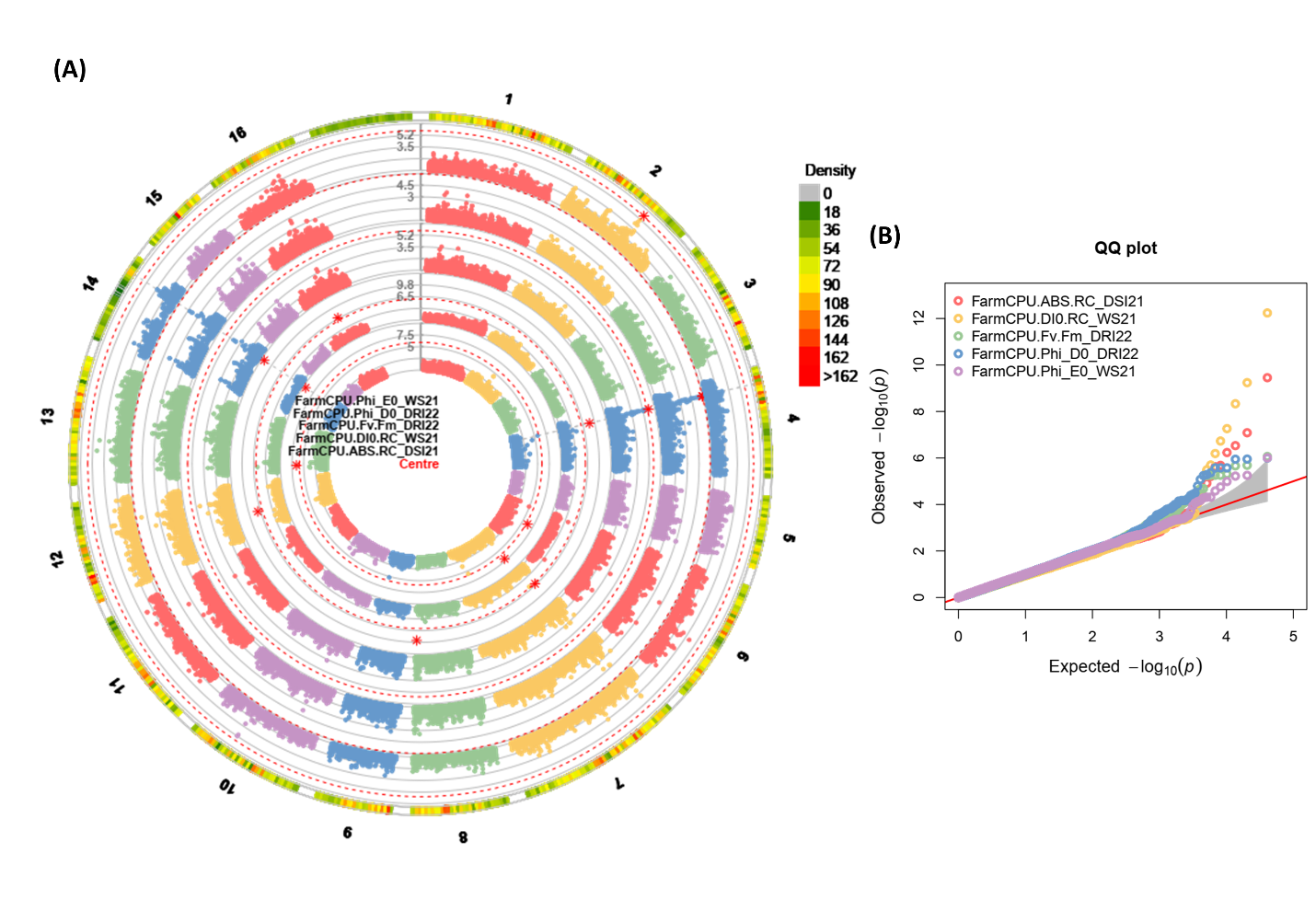


**Supplementary Figure S12.** (A) and (B) Circular Manhattan plots (left), and quantile-quantile plots (right) of association analysis using the MGBS dataset and FarmCPU (Q *+* K) model for chlorophyll fluorescence related traits from centre to the outside of plot including; (a) ABS/RC_DSI21; (b) DI_0_/RC_WS21, (c) F_V_/F_M__DRI22, (d) φ_Do__DRI22, and (e) φ_Eo__WS21. The circles of red dashed lines represent the Bonferroni-corrected significance threshold. Red stars indicate genome-wide significant findings. The numbers and colored boxes around the outermost circle indicate the chromosome and the density of single-nucleotide polymorphisms (SNPs) where green to red represent lower to higher density. Vertical grey dashed lines are drawn through GWAS findings to indicate multi-trait associations. For QQ plots, X-axis represents expected − log_10_ (*p-value*) and Y-axis is observed − log_10_ (*p-value*) of each SNPs. Water-stress: WS, drought stress index: DSI, and drought recovery index: DRI. 21: severe drought stress in the second year, and 22: recovery condition in the second year.

**
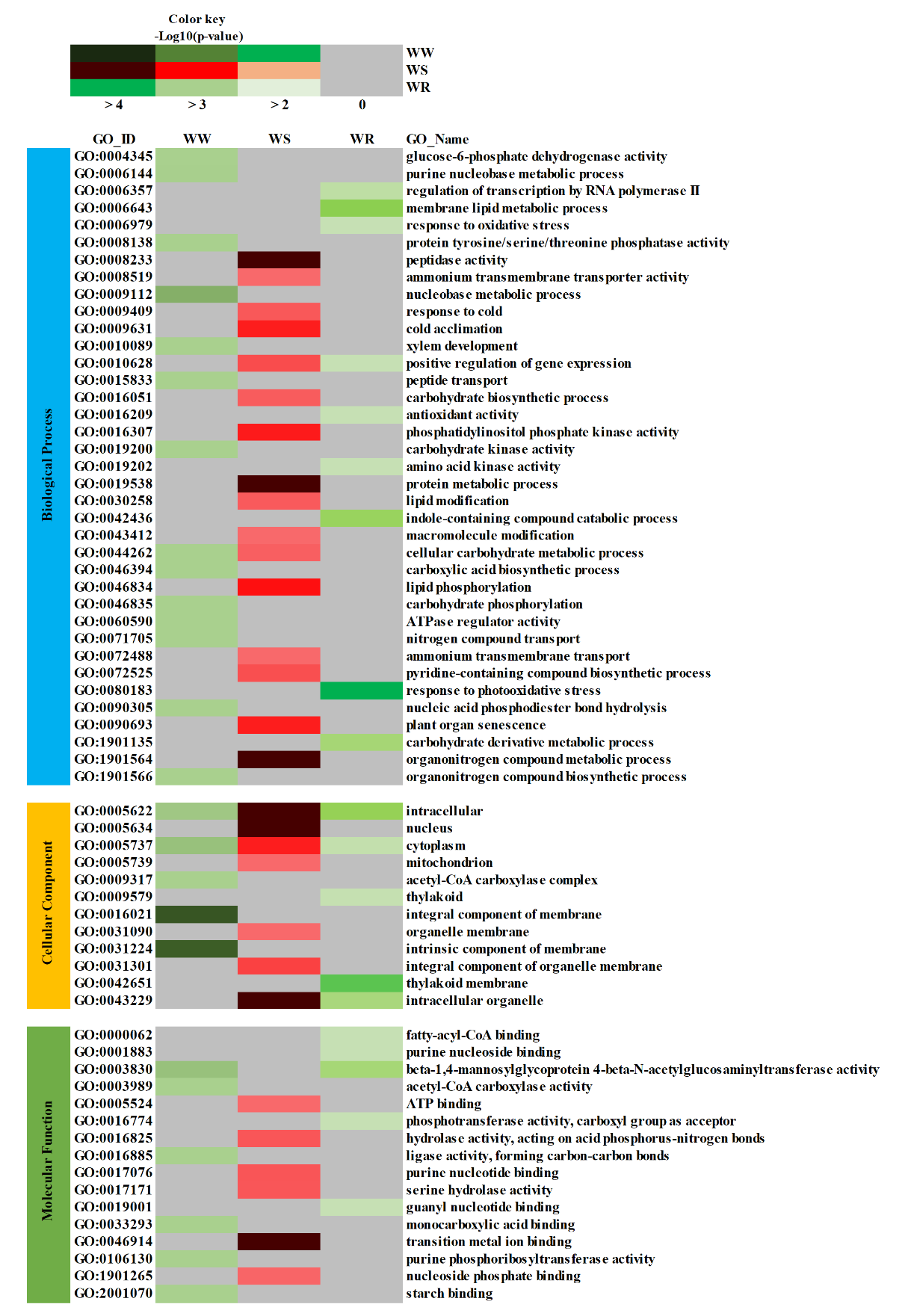
**

**Supplementary Figure S13.** Top Gene Ontology (GO) terms significantly enriched using genes associated with photosynthetic traits under well-water (WW), water-stress (WS) and water-recovery (WR) conditions**.** Different colours in X-axis represent different significant levels of the GO terms. The y-axis represents the GO terms.


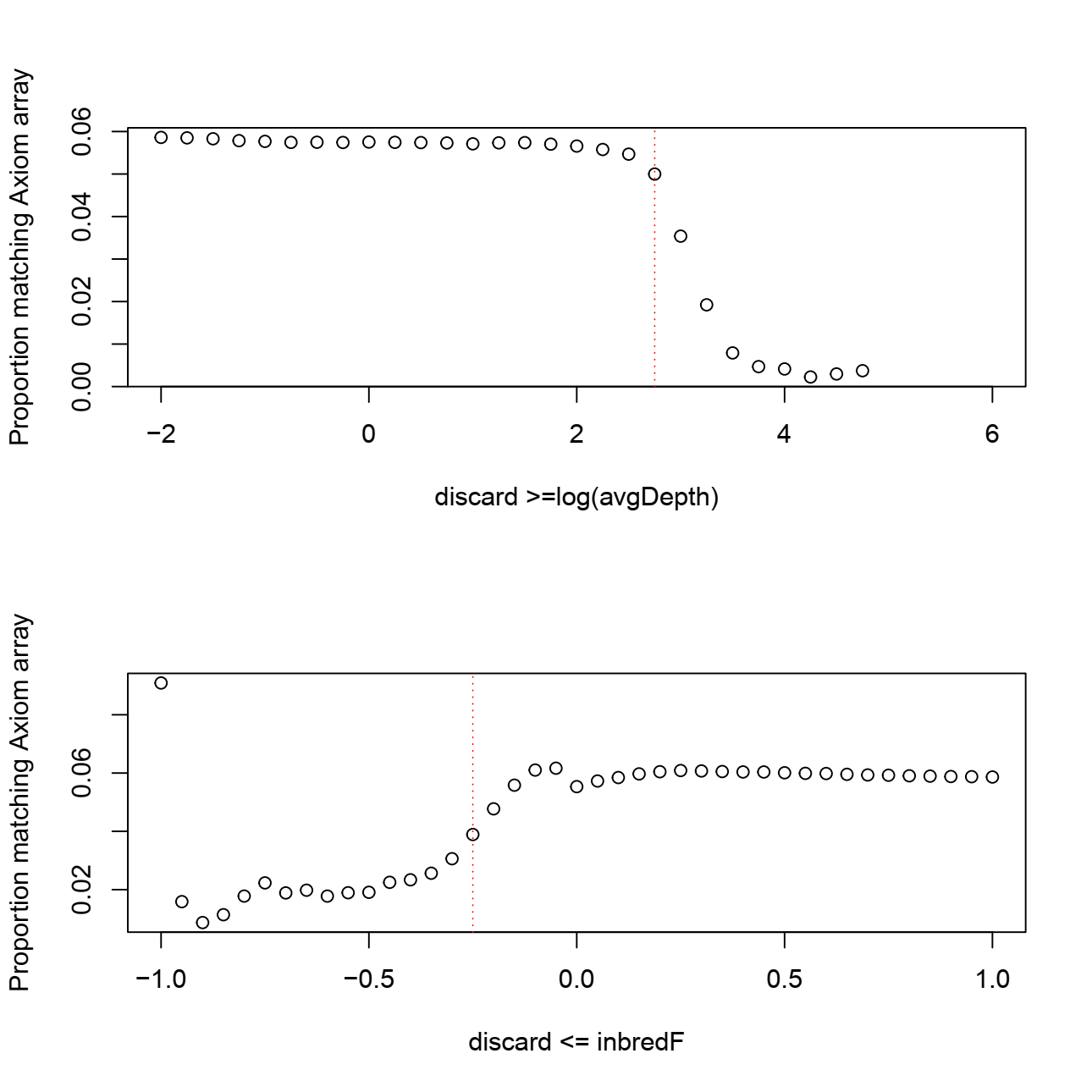


**Supplementary Figure S14.** Comparing the proportion of GBS SNPs matching the Axiom array at different thresholds.

| **Table S1.** Geographical and ecological data of the walnut populations studied | | | | | | | | |
| --- | --- | --- | --- | --- | --- | --- | --- | --- |
| **Country** | **Province** | **Region** | **Sample size** | **Altitude**  **(m)** | **Longitude**  **(E)** | **Latitude**  **(N)** | **Annual**  **rainfall (mm)** | **Annual avg. temp. (C)** |
| Iran | Kerman | Baft-Gugher | 13 | 2763 | 56°27’ | 29°31’ | 247.55 | 15.33 |
| Iran | Kerman | Rabor | 16 | 2730 | 56°57’ | 29°24’ | 267 | 15 |
| Iran | Kerman | Rabor-Hanza | 6 | 2850 | 57°12’ | 29°19’ | 267 | 15 |
| Iran | Kerman | Bardsir | 6 | 2823 | 56°29’ | 29°37’ | 171.5 | 14.6 |
| Iran | Fars | Eqlid | 8 | 2167 | 52° 47’ | 30° 54’ | 305.35 | 12.98 |
| Iran | Fars | Bavanat | 5 | 2407 | 53°31’ | 30°26’ | 209.5 | 15.85 |
| Iran | Semnan | Shahmirzad | 11 | 2061 | 53°20’ | 35°49’ | 206.24 | 12.91 |
| Iran | Ilam | Ilam | 6 | 1387 | 46°30’ | 33°40’ | 560.54 | 16.93 |
| Iran | Yazd | Taft | 18 | 2450 | 53°48’ | 31°45’ | 75.05 | 19.48 |
| Iran | Markazi | Delijan-Jasb | 15 | 2185 | 50°48’ | 34°06’ | 171.66 | 17.59 |
| Iran | West Azerbaijan | Khoy | 11 | 1479 | 44°46’ | 38°35’ | 285 | 12.57 |
| Iran | West Azerbaijan | Orumiyeh | 2 | 1016 | 48°05’ | 39°01’ | 324 | 14.53 |
| Iran | Ardabil | Germi | 2 | 1350 | 45°04’ | 37°33’ | 334 | 11.3 |
| Iran | Isfahan | Kashan | 7 | 1988 | 51°02’ | 34°07’ | 133.9 | 19.66 |
| Iran | Isfahan | Najafabad | 4 | 1682 | 51°20’ | 32°38’ | 159.74 | 18.12 |
| Iran | Isfahan | Shahreza | 2 | 2105 | 51°49’ | 32°02’ | 144.54 | 15.62 |
| Iran | Hamadan | Nahavand | 10 | 1644 | 48°25’ | 34°15’ | 375 | 14.58 |
| Iran | Qazvin | Qazvin | 3 | 1620 | 50°25’ | 36°21’ | 313.77 | 14.34 |
| Iran | Kurdistan | Baneh | 1 | 1652 | 45°53’ | 36°04’ | 628.75 | 14.3 |
| Iran | Kurdistan | Saqqez | 1 | 1798 | 46°37’ | 36°00’ | 474.33 | 12.09 |
| Iran | Kohgiluyeh and Boyer-Ahmad | Yasuj | 1 | 2219 | 51°31’ | 35°03’ | 819.75 | 14.97 |
| **Reference cultivar in genotyping array** | | | | | | | | |
| USA | California | Davis | 1 | 9 | 121° 28′ W | 38° 33′ | 508 | 16.1 |

| Table S2. Summary of variance analysis for the main effects of families and withholding treatment, and their interactions on the gas-exchange and chlorophyll fluorescence parameters measured during severe water-stress in the first-year experiment | | | | | |
| --- | --- | --- | --- | --- | --- |
|  | Mean Square | | | |  |
|  | Families | Water-stress treatment | Families × Water-stress treatment | Experimental error | Coefficient of variation (CV%) |
| DF | 139 | 1 | 139 | 280 |  |
| Gas-exchange related parameters | | | | | |
| P_n_ | 11.51** | 682.77** | 3.99** | 1.27 | 33.4% |
| g_s_ | 0.0009** | 0.063** | 0.0004** | 0.0001 | 39.9% |
| WUE | 6108** | 120771** | 751** | 328 | 11.7% |
| Ci | 2191.4** | 160271.9** | 1587.2** | 736.2 | 24.2% |
| CE | 0.0005** | 0.011** | 0.0002** | 0.00007 | 29.4% |
| T_r_ | 3.44** | 161.58** | 0.95** | 0.22 | 33.7% |
| WUEi | 7.14** | 45.003** | 0.48** | 0.14 | 11.62% |
| VpdL | 4.56** | 0.46** | 0.35** | 0.02 | 2.82% |
| Ci/Ca | 0.02** | 1.29** | 0.009** | 0.0044 | 22.4% |
| Fluorescence parameters | | | | | |
| F_0_ | 0.0006** | 0.045** | 0.0004** | 0.00006 | 12.97% |
| F_V_ | 0.0008** | 0.26** | 0.001** | 0.0002 | 10.87% |
| F_M_ | 0.0011** | 0.15** | 0.0009** | 0.0003 | 10.18% |
| F_M_/F_0_ | 1.05** | 269.14** | 0.87** | 0.09 | 9.39% |
| F_V_/F_0_ | 1.22** | 236.19** | 0.81** | 0.082 | 12.39% |
| F_V_/F_M_ | 0.022** | 4.23** | 0.021** | 0.0006 | 3.53% |

* Significant at *P<*0.05, ** Significant at *P<*0.001, ns: no significant. DF= Degree of Freedom

| Table S3. Summary of variance analysis for the main effects of families and withholding treatment, and their interactions on the gas-exchange parameters measured during severe water-stress and subsequent re-watering in the second-year experiment | | | | | |
| --- | --- | --- | --- | --- | --- |
|  | Mean Square | | | |  |
|  | Families | Water-stress treatment | Families × Water-stress treatment | Experimental error | Coefficient of variation (CV%) |
| DF | 139 | 1 | 139 | 280 |  |
| Gas-exchange related parameters under severe drought stress | | | | | |
| P_n_ | 21.42** | 1051.26** | 6.85** | 1.77 | 33.7% |
| g_s_ | 0.0014** | 0.09** | 0.0006** | 0.00014 | 40.5% |
| WUE | 5641** | 154648** | 741** | 379 | 12.16% |
| Ci | 2473.5** | 221997.7** | 1749.7** | 858.6 | 25.81% |
| CE | 0.0026** | 0.01** | 0.0007** | 0.00035 | 53.9% |
| T_r_ | 3.24** | 193.77** | 1.03** | 0.26 | 34.3% |
| WUEi | 6.64** | 60.25** | 0.41** | 0.16 | 12.04% |
| VpdL | 4.49** | 0.57** | 0.31** | 0.024 | 3.4% |
| Ci/Ca | 0.02** | 1.63** | 0.009** | 0.005 | 24.52% |
| Gas-exchange related parameters under re-watering condition | | | | | |
| P_n_ | 11.68** | 605.86** | 3.03** | 1.22 | 33.44% |
| g_s_ | 0.00098** | 0.058** | 0.0003** | 0.0001 | 41.04% |
| WUE | 6281** | 113725** | 493** | 310 | 11.26% |
| Ci | 2323.8** | 153218.1** | 823.6** | 648.9 | 22.87% |
| CE | 0.0005** | 0.01** | 0.00018** | 0.00007 | 29.8% |
| T_r_ | 3.23** | 147.71** | 0.82** | 0.22 | 33.9% |
| WUEi | 7.13** | 42.12** | 0.43** | 0.13 | 11.17% |
| VpdL | 4.72** | 1.25** | 0.28** | 0.04 | 4.09% |
| Ci/Ca | 0.021** | 1.24** | 0.005** | 0.0041 | 21.5% |

* Significant at *P<*0.05, ** Significant at *P<*0.001, ns: no significant. DF= Degree of Freedom

| **Table S4.** Summary of variance analysis of chlorophyll fluorescence parameters based on a general linear model for Families (F), treatment (T) and their interactions **(**F×T) under severe water-stress in the second year experiment | | | | |
| --- | --- | --- | --- | --- |
| Phenotype category | Traits | Families | Water-stress treatment | Families × Water-stress treatment |
| Primary fluorescence measurements | F_0_ | ** | ** | ** |
|  | F_J_ | ** | ** | ** |
|  | F_I_ | ** | ** | ** |
|  | F_M_ | ** | ** | ** |
|  | F_V_ | ** | ** | ** |
|  | V_J_ | ** | ** | ** |
|  | V_I_ | ** | ns | ** |
| Relative ratios | F_M_/F_0_ | ** | ** | ** |
|  | F_V_/F_0_ | ** | ** | ** |
|  | F_V_/F_M_ | ** | ** | ** |
| Derived parameters | M_0_ | ** | ** | ** |
|  | N | ** | ** | ** |
|  | Ψ_0_ | ** | ** | ** |
|  | φ_Eo_ | ** | ** | ** |
|  | φ_Do_ | ** | ** | ** |
|  | Φ_pav_ | ** | ** | ** |
|  | PI_ABS_ | ** | ** | ** |
| Energy flux parameters | ABS/RC | ** | ** | ** |
|  | TR_0_/RC | ** | ** | ** |
|  | ET_0_/RC | ** | ** | ** |
|  | DI_0_/RC | ** | ** | ** |

* Significant at *P<*0.05, ** Significant at *P<*0.001, ns: no significant.

| **Table S5.** Summary of variance analysis of chlorophyll fluorescence parameters based on a general linear model for Families (F), treatment (T) and their interactions (F×T) under re-watering in the second year of experiment | | | | |
| --- | --- | --- | --- | --- |
| Phenotype category | Traits | Families | Water-recovery treatment | Families × Water-stress treatment |
| Primary fluorescence measurements | F_0_ | ** | ** | ** |
|  | F_J_ | ** | * | ** |
|  | F_I_ | ** | ns | ** |
|  | F_M_ | ** | ns | ** |
|  | F_V_ | ** | ** | ** |
|  | V_J_ | ** | ** | ** |
|  | V_I_ | ** | ** | ** |
| Relative ratios | F_M_/F_0_ | ** | ** | ** |
|  | F_V_/F_0_ | ** | ** | ** |
|  | F_V_/F_M_ | ** | ** | ** |
| Derived parameters | M_0_ | ** | ** | ** |
|  | N | ** | ** | ns |
|  | Ψ_0_ | ** | ** | * |
|  | φ_Eo_ | ** | ** | ** |
|  | φ_Do_ | ** | ** | ** |
|  | Φ_pav_ | ** | ** | ns |
|  | PI_ABS_ | ** | ** | ** |
| Energy flux parameters | ABS/RC | ** | ** | ** |
|  | TR_0_/RC | ** | ** | ** |
|  | ET_0_/RC | ** | * | * |
|  | DI_0_/RC | ** | ** | ** |

* Significant at *P<*0.05, ** Significant at *P<*0.001, ns: no significant.

| **Table S6.** Summary of suggestive marker-trait associations identified by GWAS analysis using two approaches (FarmCPU and MLMM) for all the photosynthetic traits across six categories (A–F) in well-water (WW), water-stress (WS), water-recovery (WR) conditions and for drought stress index (DSI) and drought recovery index (DRI)of traits as a relative measure | | | | | | |
| --- | --- | --- | --- | --- | --- | --- |
| **Trait Classification** | **Marker-Trait** | **WW** | **WS** | **WR** | **DSI** | **DRI** |
| (A) Chl fluorescence (Mother trees- Array) | Associations | 147 | 161 | 103 | 112 | 69 |
|  | Unique SNPs | 103 | 91 | 67 | 71 | 56 |
| (B) Chl fluorescence (Mother trees- GBS) | Associations | 198 | 191 | 93 | 117 | 103 |
|  | Unique SNPs | 93 | 105 | 46 | 68 | 32 |
| (C) Chl fluorescence (Progeny-GBS) | Associations | 223 | 141 | 76 | 87 | 129 |
|  | Unique SNPs | 120 | 65 | 50 | 49 | 88 |
| (D) Gas exchange (Mother trees- Array) | Associations | 115 | 69 | 18 | 43 | 21 |
|  | Unique SNPs | 73 | 57 | 11 | 31 | 16 |
| (E) Gas exchange (Mother trees- GBS) | Associations | 134 | 86 | 36 | 70 | 47 |
|  | Unique SNPs | 75 | 56 | 25 | 48 | 36 |
| (F) Gas exchange (Progeny-GBS) | Associations | 90 | 51 | 32 | 68 | 55 |
|  | Unique SNPs | 44 | 33 | 22 | 33 | 35 |
| Total associations | Associations | 907 | 699 | 358 | 497 | 424 |
|  | Unique SNPs | 508 | 407 | 221 | 300 | 263 |
| Associations detected by the FarmCPU approach | Associations | 461 | 362 | 176 | 253 | 204 |
|  | Unique SNPs | 293 | 249 | 119 | 193 | 145 |
| Associations detected by the MLMM approach | Associations | 446 | 337 | 182 | 244 | 220 |
|  | Unique SNPs | 319 | 250 | 141 | 200 | 173 |
| SNP-traits detected by both approaches | Total SNPs | 907 | 699 | 358 | 497 | 424 |
|  | Unique SNPs | 612 | 499 | 260 | 393 | 318 |

| **Table S7.** Summary of significant marker-trait associations identified by GWAS analysis using two approaches (SNP-array and GBS) for all the photosynthetic traits across all conditions (WW, WS, WR, DSI and DRI) | | | | |
| --- | --- | --- | --- | --- |
| **Genotyping method/Trait** | **Mother Array** | **Mother GBS** | **Progeny GBS** | **Total** |
| F_V_ | 29 | 15 | 3 | 47 |
| PC3 | 10 | 21 | 11 | 42 |
| DI_0_/RC | 21 | 8 | 11 | 40 |
| F_M_/F_0_ | 15 | 16 | 9 | 40 |
| PI_ABS_ | 11 | 9 | 20 | 40 |
| F_V_/F_0_ | 15 | 13 | 9 | 37 |
| PC2 | 14 | 13 | 9 | 36 |
| N | 15 | 10 | 11 | 36 |
| RWC | 31 | 3 | 1 | 35 |
| F_V_/F_M_ | 12 | 9 | 11 | 32 |
| P_n_ | 10 | 17 | 1 | 28 |
| φ_D0_ | 8 | 10 | 9 | 27 |
| F_M_ | 4 | 13 | 9 | 26 |
| PC1 | 5 | 7 | 14 | 26 |
| ψ_0_ | 7 | 9 | 9 | 25 |
| CE | 4 | 19 | 2 | 25 |
| F_I_ | 6 | 3 | 16 | 25 |
| ABS/RC | 16 | 8 |  | 24 |
| T_r_ | 20 |  | 1 | 21 |
| Trans | 21 |  |  | 21 |
| gs | 19 | 2 |  | 21 |
| TR_0_/RC | 6 | 7 | 8 | 21 |
| φ_E0_ | 1 | 12 | 7 | 20 |
| WUEi | 15 | 1 | 3 | 19 |
| V_J_ | 7 | 9 | 2 | 18 |
| M_0_ | 7 | 9 | 1 | 17 |
| F_J_ |  | 11 | 6 | 17 |
| F_0_ | 5 |  | 3 | 8 |
| WUE | 5 | 2 |  | 7 |
| C_i_ |  | 5 |  | 5 |
| DS |  |  | 4 | 4 |
| φ_Pav_ | 1 |  | 2 | 3 |
| ET_0_/RC |  | 2 |  | 2 |
| Total Association | **340** | **263** | **192** | **795** |
| Total Unique SNPs | **228** | **198** | **152** | **578** |

| **Table S8.** Summary of suggestive marker-trait associations identified by GWAS analysis using two approaches (SNP-array and GBS) for all the photosynthetic traits across all conditions (WW, WS and WR) and phenotypic plasticity of traits (DSI and DRI) | | | | |
| --- | --- | --- | --- | --- |
| **Genotyping**  **method** | **Mother**  **Array** | **Mother**  **GBS** | **Progeny**  **GBS** | **Total** |
| F_V_ | 77 | 47 | 53 | 177 |
| F_V_/F_M_ | 60 | 53 | 25 | 138 |
| CE | 30 | 40 | 63 | 133 |
| F_V_/F_0_ | 58 | 40 | 31 | 129 |
| PI_ABS_ | 32 | 58 | 28 | 118 |
| PC3 | 24 | 46 | 48 | 118 |
| PC2 | 38 | 32 | 47 | 117 |
| F_M_/F_0_ | 51 | 38 | 25 | 114 |
| RWC | 63 | 12 | 31 | 106 |
| DI_0_/RC | 38 | 38 | 24 | 100 |
| F_M_ | 30 | 40 | 30 | 100 |
| PC1 | 26 | 45 | 25 | 96 |
| φ_E0_ | 30 | 26 | 40 | 96 |
| F_0_ | 29 | 43 | 20 | 92 |
| g_s_ | 34 | 22 | 36 | 92 |
| φ_D0_ | 38 | 28 | 20 | 86 |
| P_n_ | 35 | 12 | 32 | 79 |
| WUEi | 34 | 31 | 11 | 76 |
| N | 36 | 19 | 19 | 74 |
| Trans | 43 | 24 | 6 | 73 |
| ABS/RC | 37 | 11 | 23 | 71 |
| F_I_ | 28 | 26 | 16 | 70 |
| T_r_ | 38 | 24 | 5 | 67 |
| WUE | 19 | 24 | 23 | 66 |
| ψ_0_ | 22 | 27 | 16 | 65 |
| V_J_ | 23 | 17 | 16 | 56 |
| F_J_ | 16 | 14 | 22 | 52 |
| ET_0_/RC | 15 | 8 | 29 | 52 |
| V_I_ | 9 | 2 | 40 | 51 |
| C_i_ | 18 | 20 | 11 | 49 |
| TR_0_/RC | 13 | 19 | 16 | 48 |
| M_0_ | 15 | 16 | 17 | 48 |
| φ_Pav_ | 5 | 26 | 8 | 39 |
| C_i_.C_a_ | 11 | 10 | 2 | 23 |
| DS |  | 14 |  | 14 |
| Total Association | **1075** | **952** | **858** | **2885** |
| Total Unique SNPs | **544** | **524** | **481** | **1543** |
